# Supplementary material for: A moth odorant receptor highly expressed in the ovipositor is involved in detecting host-plant volatiles
Source: eLife. 2020 May 21;9:e53706. doi: 10.7554/eLife.53706 (PMC7308088; doi:10.7554/eLife.53706)
Supplement: Supplementary file 1. — OR, odorant receptor; GR, gustatory receptor; IR, antennal ionotropic receptor; iGluR, ionotropic glutamate receptor. [file elife-53706-supp1.docx]

**Supplementary file 1.** Expression values of putative chemosensory receptors in the pheromone gland-ovipositor complex of *H. assulta* (*Hass*) and *H. armigera* (Harm)*.* OR, odorant receptor; GR, gustatory receptor; IR, antennal ionotropic receptor; iGluR, ionotropic glutamate receptor.

|  | Putative Chemosensory Receptors | TPM | Putative Chemosensory Receptors | TPM |
| --- | --- | --- | --- | --- |
| ***OR*** | ***HassOR31*** | 21.257 | *HarmOR26* | 8.757 |
|  | *HassOR30* | 0.989 | *HarmOR42* | 1.239 |
|  | ***HassORco*** | 0.878 | *HarmOR47* | 1.163 |
|  | *HassOR44* | 0.588 | *HarmOR9* | 0.232 |
|  | *HassOR48* | 0.457 | *HarmOR46* | 0.122 |
|  | *HassOR45* | 0.215 | ***HarmOR31*** | 0.104 |
|  | *HassOR11* | 0.205 | *HarmOR4* | 0.099 |
|  | *HassOR39* | 0.196 | *HarmOR45* | 0.058 |
|  | *HassOR20* | 0.159 | ***HarmORco*** | 0.035 |
|  | *HassOR46* | 0.159 | *HarmOR50* | 0.035 |
|  | *HassOR47* | 0.159 |  |  |
|  | *HassOR52* | 0.122 |  |  |
|  | *HassOR27* | 0.112 |  |  |
|  | *HassOR41* | 0.112 |  |  |
|  | *HassOR10* | 0.103 |  |  |
|  | *HassOR4* | 0.103 |  |  |
|  | *HassOR9* | 0.103 |  |  |
|  | *HassOR50* | 0.075 |  |  |
|  | *HassOR26* | 0.065 |  |  |
|  | *HassOR29* | 0.065 |  |  |
|  | *HassOR40* | 0.047 |  |  |
|  | *HassOR42* | 0.047 |  |  |
| ***GR*** | *HassGR9* | 0.886 | *HarmGR4* | 6.79 |
|  | *HassGR108* | 0.522 | *HarmGR3* | 0.139 |
|  | *HassGR180* | 0.317 | *HarmGR9* | 0.11 |
|  | *HassGR14* | 0.233 |  |  |
|  | *HassGR8* | 0.112 |  |  |
|  | *HassGR67* | 0.0929 |  |  |
| ***IR*** | *HassIR76b* | 6.653 | *HarmIR68a* | 2.096 |
|  | *HassIR7d.2* | 3.696 | *HarmIR64a* | 1.448 |
|  | *HassIR7d.3* | 1.148 | *HarmIR98a* | 0.035 |
|  | *HassIR75d* | 0.709 |  |  |
|  | *HassIR25a* | 0.476 |  |  |
|  | *HassIR143* | 0.439 |  |  |
|  | *HassIR75p.2* | 0.242 |  |  |
|  | *HassIR8a* | 0.149 |  |  |
|  | *HassIR93a* | 0.14 |  |  |
|  | *HassIR1.1* | 0.084 |  |  |
|  | *HassIR7d.1* | 0.075 |  |  |
|  | *HassIR21a* | 0.056 |  |  |
|  | *HassIR75q.2* | 0.037 |  |  |
| ***iGluR*** | *HassiGluR7* | 23.049 | *HarmiGluR5* | 33.849 |
|  | *HassiGluR6* | 16.965 | *HarmiGluR7* | 9.116 |
|  | *HassiGluR12* | 11.263 | *HarmiGluR6* | 3.016 |
|  | *HassiGluR9* | 6.943 | *HarmiGluR2* | 0.961 |
|  | *HassiGluR2* | 5.58 | *HarmiGluR8* | 0.208 |
|  | *HassiGluR10* | 2.314 | *HarmiGluR4* | 0.133 |
|  | *HassiGluR4* | 0.951 |  |  |
|  | *HassiGluR8* | 0.149 |  |  |
|  | *HassiGluR3* | 0.037 |  |  |
