# Supplementary material for: A moth odorant receptor highly expressed in the ovipositor is involved in detecting host-plant volatiles
Source: eLife. 2020 May 21;9:e53706. doi: 10.7554/eLife.53706 (PMC7308088; doi:10.7554/eLife.53706)
Supplement: Supplementary file 2. [file elife-53706-supp2.docx]

**Supplementary file 2.** Primers used for qRT-PCR, RT-PCR, probe synthesis and full-length cDNA cloning. F: forward strand; R: reverse strand

| gene | primer | sequence（5’ – 3’） |
| --- | --- | --- |
| *HassOR31* | qRT-PCR/F | CAGTGTGAAGAAGTGGTGCG |
|  | qRT-PCR/R | GCATTAGCGAGCACATGACC |
|  | RT-PCR/F | AAGCGTCGTCTGTATCGT |
|  | RT-PCR/R | TCCAACAACCAGGTGAAT |
|  | XhoI-F | CTCGAG*GCCACC*ATGAATTCAATAC |
|  | XbaI-R | TCTAGACTATTTTCTCAGCAGTGTGTAATAAC |
|  | probe- F | GAACCTAGAAGACCCAGACCG |
|  | probe- R | GCACAAAGCATTAGCGAGCA |
| *HassORco* | EcoRI -F | GAATTC*GCCACC*ATGATGACCAAAG |
|  | XhoI -R | CTCGAGTTACTTGAGTTGTACCAACAC |
|  | probe- F | TGAGGTCAACGAACTGACGG |
|  | probe- R | GTACCATACGTGTCGCCGAT |
| *HassiGluR7* | EcoRI -F | GAATTC*GCCACC*ATGTGTTGGTA |
|  | XhoI -R | CTCGAGTCAAACAGCTTTTTCCA |
| *Hass18S* | qRT-PCR/F | CGTTGCTGGGAAGTTGACCA |
|  | qRT-PCR/R | CTTCCGCAGGTTCCCCTACG |
| *HassActin* | RT-PCR/F | ACCAACTGGGACGACATGGAG |
|  | RT-PCR/R | CGTCAGGATCTTCATGAGGTAGTC |

Letters with underline indicates restriction enzyme sites. Italic letters represent the Kozak sequence which is used to enhance the translation efficiency.
