# Supplementary material for: A moth odorant receptor highly expressed in the ovipositor is involved in detecting host-plant volatiles
Source: eLife. 2020 May 21;9:e53706. doi: 10.7554/eLife.53706 (PMC7308088; doi:10.7554/eLife.53706)
Supplement: Supplementary file 3. [file elife-53706-supp3.docx]

**Sup****plementary file** **3.** Tested compounds for functional analysis of HassOR31.

| compounds | purity | supplier |
| --- | --- | --- |
| (+)-3-Carene | 99% | Fluka |
| (±)-Linalool | 97% | Fluka |
| **(−)-E-Caryophyllene** | 99% | Fluka |
| (−)-α-Pinene | 98% | Aldrich |
| **(S)-(-)-Limonene** | 96% | Aldrich |
| 1-Butanol | 99.80% | Sigma |
| 1-Hexanol | 99% | Fluka |
| **1-Octanol** | 99% | Aldrich |
| 1-Pentanol | 99% | Fluka |
| 2-Hexanol | 99% | Aldrich |
| 2-Methyl-1-butanol | 99% | Aldrich |
| **2-Phenylethanol** | 99% GC | Sigma |
| 3-Hexanol | 98% | Roth |
| 3-Methyl-1-butanol | 99% | Sigma |
| Anethol | 99% | Aldrich |
| **Benzaldehyde** | 98% | Aldrich |
| Benzyl alcohol | 98% | Aldrich |
| Borneol | 97% | Aldrich |
| Z-3-Hexenol-1 | 98% | Roth |
| **Z-3-Hexenyl 2-methyl butyrate** | 97+% | SAFC |
| **Z-3-Hexenyl acetate** | 97% | Roth |
| **Z-3-Hexenyl butyrate** | 98+% | Sigma |
| Z-3-Hexenyl salicylate | 97% | Aldrich |
| Z-6-Nonenol | 95% | Aldrich |
| **Z-Jasmone** | 85% | Aldrich |
| **Citral** | 96% | Aldrich |
| E-β-farnesene | 90+% | Sigma |
| Farnesene isomer (Farnesene) | 98+% | SAFC |
| Farnesol | 95% | Aldrich |
| **Geraniol** | 96% | Fluka |
| Guaiene | 96% | Aldrich |
| Heptan-1-ol | 100% | Fluka |
| Methyl benzoate | 99% | Aldrich |
| Methyl salicylate | 99%GC | Sigma |
| **Myrcene** | 90% | Aldrich |
| **Nonanal** | 97% | Aldrich |
| **Ocimene iosmers** | 90+% | Aldrich |
| Phenylacetaldehyde | 90+% | Sigma |
| Salicylaldehyde | 98% | Aldrich |
| E-2-Hexenal | 97% | Fluka |
| E-2-Hexenyl acetate | 98% | Aldrich |
| E-Hexen-2-ol-1 | 95% | Fluka |
| E-Hexen-3-ol-1 | 95% | Roth |
| Verbenol | 95% | Aldrich |
| **Z11-16:Ald** | 95% | Shin-Etsu |
| **Z9-16:Ald** | 95% | Shin-Etsu |
| **α--Phellandrene** | 85% | Aldrich |
| α-Terpinene | 92% | Roth |
| β-Pinene | 99% | Roth |

Compounds in bold were used in SSR experiments.
